# Increased expression of surface CD44 in hypoxia-DCs skews helper T cells toward a Th2 polarization

**DOI:** 10.1038/srep13674

**Published:** 2015-09-01

**Authors:** Meixiang Yang, Yanguo Liu, Guangwen Ren, Qianqian Shao, Wenjuan Gao, Jintang Sun, Huayang Wang, Chunyan Ji, Xingang Li, Yun Zhang, Xun Qu

**Affiliations:** 1 Institute of Basic Medical Sciences, Qilu Hospital, Shandong University, Jinan, 250012, Shandong, China; 2 Department of Medical Oncology, Qilu Hospital, Shandong University, Jinan, 250012, Shandong, China; 3 Department of Molecular Biology, LTL255, Washington Road, Princeton University, Princeton, New Jersey 08544, USA.; 4 Department of Hematology, Qilu Hospital, Shandong University, Jinan, 250012, Shandong, China; 5 Department of Neurosurgery, Key Laboratory of Neuro-Oncology and Immunology, Qilu Hospital, Shandong University, Jinan, 250012, Shandong, China; 6 The Key Laboratory of Cardiovascular Remodelling and Function Research, Chinese Ministry of Education and Chinese Ministry of Health, Qilu Hospital, Shandong University, Jinan, Shandong 250012, China

## Abstract

A low partial oxygen pressure (hypoxia) occurs in many pathological environments, such as solid tumors and inflammatory lesions. Understanding the cellular response to hypoxic stress has broad implications for human diseases. As we previously reported, hypoxia significantly altered dendritic cells (DCs) to a DC2 phenotype and promoted a Th2 polarization of naïve T cells with increased IL-4 production. However, the underlying mechanisms still remain largely unknown. In this study, we found the over-expression of surface CD44 in DCs was involved in this process via ligand binding. Further investigation showed hypoxia could reduce the surface expression of membrane type 1 metalloprotease (MT1-MMP) via down-regulating the kinesin-like protein KIF2A, which subsequently alleviated the shedding of CD44 from DCs. Moreover, KIF2A expression was found negatively regulated by HIF-1α in hypoxic microenvironment. These results suggest a previously uncharacterized mechanism by which hypoxia regulates the function of DCs via KIF2A/MT1-MMP/CD44 axis, providing critical information to understand the immune response under hypoxia.

Hypoxia results from the imbalance between cellular oxygen supply and consumption and is a characteristic feature of many physiological and pathological circumstances, such as inflammation, tumor and blastocyst implantation^1^,^2^,^3^. DCs are a heterogeneous family of professional antigen presenting cells (APCs) involved in the initiation of immunity. They infiltrate into the lesions in inflammatory diseases and some solid tumors, playing an important role in the initiation, polarization and termination of the adaptive immunity^4^. After capturing antigens in the periphery, DCs migrate to primary lymphoid organs, stimulate and sensitize naïve T cells and regulate T cell responses to different polarizations^5^. On the basis of their cytokine production profiles, activated naïve CD4^+^ T cells differentiate into several subsets, including Th1 and Th2 cells^6^. Th1 cells mainly secrete IFN-γ and IL-2, whereas Th2 cells produce a variety of cytokines, including IL-4 and IL-10. Hypoxia has been reported to impact the differentiation and function of immune cells, such as T and B lymphocytes^7^,^8^,^9^. We and other groups have indicated that hypoxia also influenced DCs via regulating their differentiation toward a Th2 polarizing phenotype with increased secretion of IL-4^10^,^11^,^12^,^13^, and the phenomenon was partially mediated by hypoxia induced alteration of adenosine metabolism and expression of its binding receptor A2b^14^. However, the expression or function of a broad spectrum of genes is impacted in response to hypoxia^15^, and thus there might be other molecules or pathways involved in hypoxia-DCs primed Th2 polarization.

CD44 is the major receptor for hyaluronic acid (HA) and its expression has been found in various cell types, such as human monocytes, Langerhans cells and T cells, involved in many pathophysiological processes owing to its dual role in both cell adhesion and signaling regulation^16^,^17^,^18^,^19^,^20^,^21^. High level of CD44 has also been detected in mature DCs and suggested to play an important role in DCs-T cell interaction and further T cell activation^22^,^23^,^24^. Shedding from the cell surface is a key regulatory event for CD44 expression and this process (the proteolytic release of ectodomains) is controlledy by different proteinases, including MT1-MMP^25^,^26^,^27^. However, the role of CD44 in DCs’ function under hypoxic condition and the involved molecules regulating CD44 shedding remain undefined.

The kinesin superfamily proteins (KIFs) have important function in cell mitosis, meiosis and transport of cargo proteins^28^,^29^,^30^. Wiesner C *et al.* reported that another kinesin family protein KIF3 played an important role in MT1-MMP surface exposure and extracellular matrix degradation in macrophages^31^. However, whether MT1-MMP is regulated by the kinesin proteins, as well as their exact functions in human monocyte-derived DCs, remains unknown.

Herein we reported a novel mechanism involved in hypoxia-DCs primed Th2 polarization. We found that only KIF2A of kinesin family in DCs was significantly down-regulated by hypoxia through HIF-1α, which drove MT1-MMP surface exposure and further CD44 shedding. Our results indicated that the KIF2A/MT1-MMP/CD44 axis impelled hypoxic DCs to mediate Th2 polarization from naïve T cells. These data implicated a fundamental mechanism controlling the Th1/Th2 differentiation under hypoxia condition via DCs.

## Results

### CD44 was elevated by hypoxia in mature DCs and promoted the polarization of naïve CD4^+^ T cells toward a Th2 phenotype

**Figure 1:**
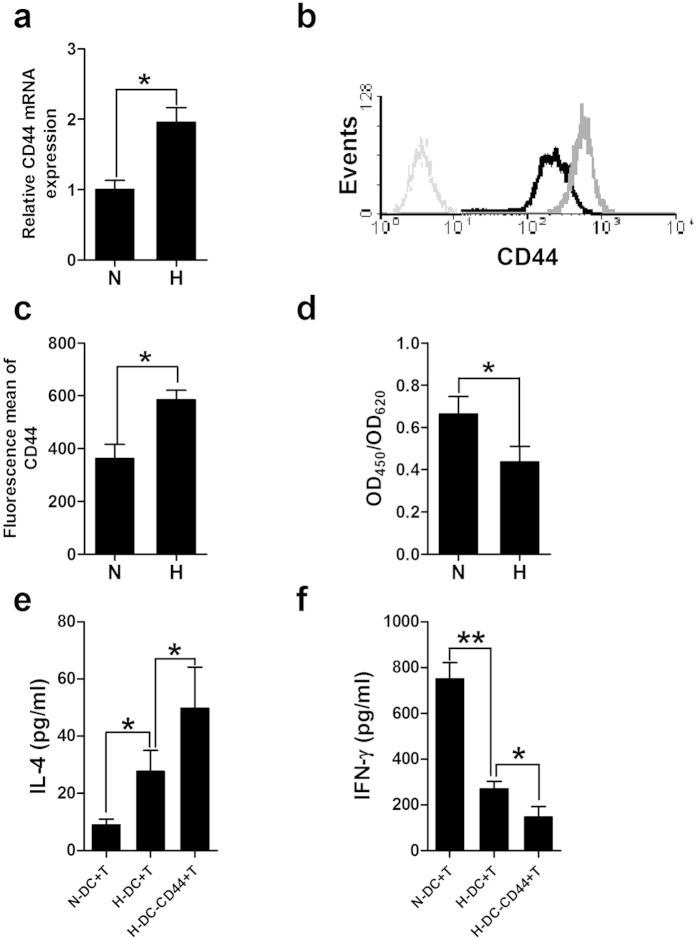
The surface expression of CD44 was elevated in mDCs under hypoxia and further promoted hypoxic DCs-mediated Th2 polarization. (**a**) The mRNA expression of CD44 in mDCs cultured under normoxia (N) or hypoxia (H) was evaluated by qRT-PCR. Results are shown as fold changes relative to CD44 mRNA levels in normoxic mDCs and represent the average of three independent experiments. (**b**,**c**) Cell samples were immunostained and analyzed by flow cytometry. (**b**) Isotype control (grey dotted line) and the surface stained CD44 on normoxic DCs (dark bold line) and hypoxic DCs (grey bold line) are presented. (**c**) The fluorescence mean of CD44 for normoxic (N) or hypoxic (H) mDCs of three separate experiments was shown. (**d**) Cell culture supernatants were harvested from each group and the shed CD44 protein was measured using the Quantikine ELISA kit. (**e**, **f**) imDCs were matured with LPS under hypoxic conditions in the presence of control IgG or anti-CD44 mAb for 48 hours. DCs matured by LPS under normoxia were also used as control. Naïve CD4^+^ T cells were polarized by cultured with mDCs. IL-4 (**e**) and IFN-γ (**f**) production was induced by adding PMA and ionomycin on day 9 and analyzed using ELISA kits. Results are shown as the mean ± SD of three different experiments. *P < 0.05, **P < 0.01.

DCs activate naïve CD4^+^ T cells and regulate their differentiation into Th1 or Th2 cells. In an earlier study, we reported that hypoxia (1% O_2_) altered human monocyte-derived DCs to a DC2 phenotype by LPS maturation and hypoxia-DCs skewed polarization of T cells toward a Th2 phenotype^12^; however, the mechanisms accounting for this were not clear. CD44 is a critical multi-functional molecule expressed in DCs and is involved in DC and T cell interactions^24^. Therefore, we analyzed CD44 expression and found its mRNA was significantly up-regulated in mature DCs (mDCs) under hypoxia by real-time PCR (Fig. 1a). Furthermore, the surface expression of CD44 in hypoxic mDCs was significantly increased compared to the normoxic mDCs (Fig. 1b), while other CD markers, including CD80 and CD86, were not affected by hypoxia as we previously reported^12^. The mean fluorescence intensity of CD44 increased up to approximately 2 folds in mDCs cultured under hypoxia (Fig. 1c), which was further corroborated by the result from ELISA that the soluble-form of CD44 in the supernatant of hypoxic DCs was significantly reduced (Fig. 1d).

Consistent with our previous findings, hypoxic DCs did polarize allogeneic CD4^+^ T cells into Th2 response with increased IL-4 and decreased IFN-γ production compared to normoxic DCs (Fig. 1e,f). To investigate whether the elevated surface expression of CD44 in hypoxic mDC was involved in T cell polarization, a mAb for CD44 (J173, Immunotech) was adopted, which caused a higher CD44 signaling through its binding to the CD44^22^. DCs were treated with immobilized anti-CD44 mAb (40 ng/ml) during LPS maturation under hypoxia and their ability to prime allogeneic T-cell polarization was further detected. T cells co-cultured with anti-CD44 treated hypoxic DCs produced less IFN-γ but more IL-4 than control hypoxic DCs (Fig. 1e,f), indicating the polarization of T cells towards a Th2 phenotype. These findings provided evidences that CD44 skewed DCs to prime naïve T cells towards a Th2 polarization.

### Decreased MT1-MMP expression in hypoxia-DCs was involved in shedding of surface CD44 from mDCs

**Figure 2:**
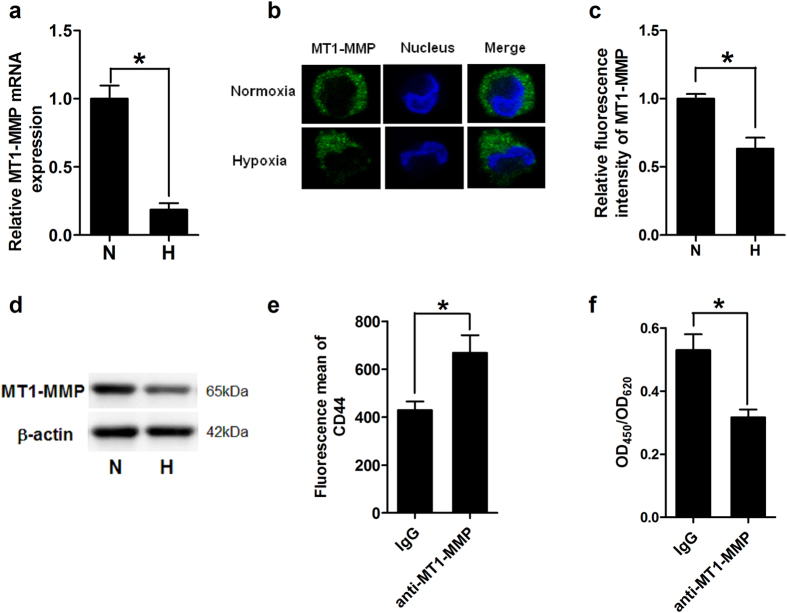
MT1-MMP was involved in regulating the surface expression of CD44 in LPS induced mature DCs. (**a**) MT1-MMP mRNA expression in mDCs cultured under normoxia (N) or hypoxia (H) was evaluated by qRT-PCR. Results are shown as fold changes relative to CD44 mRNA levels in normoxic mDCs and represent the average of three independent experiments. (**b**) Micrographs of mature DCs stained for endogenous MT1-MMP using specific primary and FITC-labeled secondary antibody, and stained for nucleus using DAPI. The location of MT1-MMP was detected using immunofluorescence microscopy (×400). (**c**) Relative fluorescence intensity of MT1-MMP in mDCs under normoxia (N) and hypoxia (H) was measured by Image J software and results are expressed as fold relative to MT1-MMP expression in normoxic mDCs (equal to 1). Data are expressed as the mean ± SD of three independent experiments. (**d**) Expression of MT1-MMP protein in mDCs was detected by western blotting. Total proteins prepared from normoxic (N) or hypoxic (H) mDCs were separated by SDS-polyacrylamide gel electrophoresis and immunoblotted with anti-MT1-MMP mAb or by parallel with β-actin antibody as a loading control. (**e**,**f**) imDCs were matured with LPS in the absence or presence of anti-MT1-MMP mAb for 48 hours. (**e**) Cell samples were immunostained and analyzed by flow cytometry. The fluorescence mean of surface stained CD44 on control IgG treated mDCs or anti-MT1-MMP treated mDCs are presented. (**f**) Cell culture supernatants were harvested from each group and the shed CD44 protein was measured using the ELISA kit. Data are expressed as the mean ± SD of four independent experiments in Fig. 2e,f. *P < 0.05.

Shedding of CD44 from cell surface is closely controlled by MT1-MMP^32^, we therefore explored whether hypoxia regulated CD44 expression in DCs through MT1-MMP. Firstly, the effect of hypoxia on MT1-MMP expression was investigated. As expected, hypoxic mDCs expressed a lower level of MT1-MMP compared with the normoxic mDCs validated by real-time PCR (Fig. 2a). Subsequently, we confirmed the appearance of MT1-MMP throughout the cytoplasm with high concentrations in the periphery or the leading edge of spontaneously migrating cells by immunofluorescence microscopy and observed relatively weaker expression of MT1-MMP in hypoxic mDCs (Fig. 2b,c). Also further confirmation was acquired by western blotting results (Fig. 2d).

Next, we investigated whether MT1-MMP was involved in the regulation of CD44 in hypoxia-DCs. For this purpose, the immature DCs (imDCs) were treated with LPS in the presence of control IgG or blocking mAb against MT1-MMP (10 μg/ml) for 48 hours under hypoxia. The results showed that the surface expression of CD44 was significantly increased in hypoxic mDCs treated with anti-MT1-MMP mAb (Fig. 2e). Consistently, blocking of MT1-MMP led to reduced level of shed CD44 in the culture supernatants from ELISA results (Fig. 2f). Taken together, these results suggested that hypoxia not only down-regulated the cell exposure of MT1-MMP, but also further increased the surface expression of CD44 in mDCs through MT1-MMP.

### Hypoxia down-regulated the expression of KIF2A in mDCs and KIF2A was involved in the cell surface exposure of MT1-MMP in mDCs

**Figure 3:**
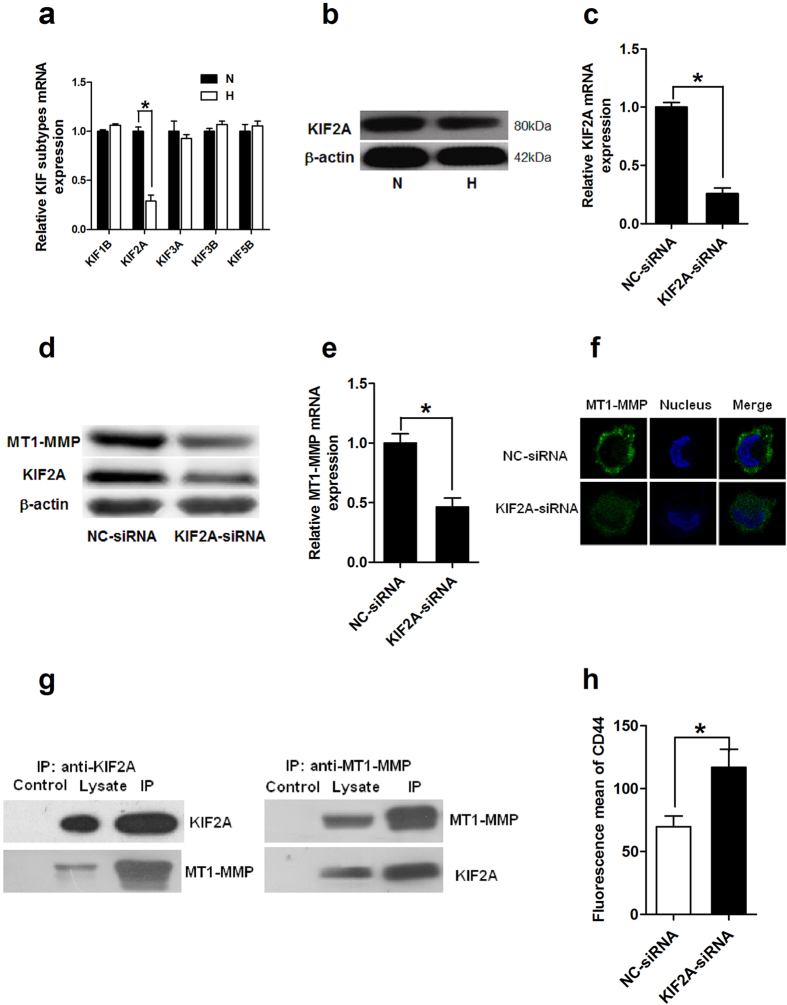
The expression of KIF2A was inhibited by hypoxia and KIF2A regulated MT1-MMP cell exposure in human monocyte-derived mDCs. (**a**) Total RNA was isolated from mDCs cultured under normoxia (N) or hypoxia (H). KIF subtypes mRNA expression was evaluated by qRT-PCR. Results are shown as fold changes relative to gene expression levels in normoxic mDCs. These results are representative of three different experiments. (**b**) Expression of KIF2A protein in mature DCs was detected by western blotting. Total proteins prepared from DCs cultured under normoxia (N) or hypoxia (H) were separated by SDS-polyacrylamide gel electrophoresis and immunoblotted with anti-KIF2A mAb. The expression of β-actin served as an internal control. (**c**–**f**) imDCs was either electroporated with KIF2A siRNA or negative control (NC) siRNA and LPS was added to induce DCs maturation for 48 hours. (**c**,**e**) KIF2A (**c**) and MT1-MMP (**e**) mRNA expression in mDCs was determined by qRT-PCR. The results are expressed as fold of mRNA levels of DCs treated with negative control siRNA (arbitrarily defined as 1) for three separate experiments. (**d**) Whole cell protein was prepared and analyzed by western blotting with indicated antibody. (**f**) DCs were stained for endogenous MT1-MMP using specific primary and FITC-labeled secondary antibody. The nucleus was stained using DAPI. The location of MT1-MMP was detected using immunofluorescence microscopy (×400). (**g**) Endogenous KIF2A or MT1-MMP from mDCs cell lysates was immunoprecipitated and the precipitates were screened for the presence of KIF2A and MT1-MMP by immunoblotting. (**h**) Cell samples were immunostained and analyzed by flow cytometry; and the fluorescence mean for each group of three separate experiments was shown in the histogram. *P < 0.05.

Our previous results showed CD44 was regulated by MT1-MMP in mDC and promoted hypoxic mDC-mediated Th2 polarization. It is reported motor proteins of the kinesin family (KIF5B and KIF3A/KIF3B) drive surface exposure of MT1-MMP and shedding of CD44 in macrophages^31^. Interestingly, by microarray analysis, one kinesin family protein-KIF2A was always found significantly down-regulated in mDCs by hypoxia, although other members of kinesins remained unchanged or undetectable (Tables 1 and 2). The expression of different subtypes of kinesins was further confirmed by real-time PCR (Fig. 3a). Moreover, the results from western blotting also showed that hypoxia could down-regulate the expression of KIF2A in mDCs (Fig. 3b). Together, these results indicated that KIF2A might be involved in the functions of mDCs under hypoxia. Therefore, we explored such possibility in the following studies.

Firstly, we investigated the effect of KIF2A on the cell surface exposure of MT1-MMP in human monocyte-derived mDCs. SiRNA specific for KIF2A was generated and evaluated for its effect on the cell surface exposure of MT1-MMP. 48 hours later after siRNA transfection, cells were collected and the expression of KIF2A was detected by real-time PCR and western blotting. A significant knockdown of KIF2A at both mRNA (Fig. 3c) and protein level (Fig. 3d, middle lane) was verified in transfected mDCs. Importantly, the expression of MT1-MMP was significantly suppressed in KIF2A-knockdown mDCs (Fig. 3e,d (upper lane)) at both mRNA and protein levels, which was further confirmed by immunofluorescence microscopy showing that surface expression of MT1-MMP was clearly alleviated in the absence of KIF2A (Fig. 3f). Moreover, we examined the association of KIF2A with MT1-MMP using immunoprecipitations. The result revealed that a reciprocal co-immunoprecipitation of endogenous KIF2A and MT1-MMP occurred in mDCs (Fig. 3g). We also explored whether down-regulation of KIF2A was associated with the expression/shedding of CD44. The results indicated that the cell surface expression of CD44 was significantly increased in KIF2A-repressed mDCs (Fig. 3h). Collectively, these data indicated that KIF2A was involved in regulating the surface exposure of MT1-MMP and CD44 expression in mDCs, which suggests that hypoxia might support mDCs primed Th2 responses through down-regulation of KIF2A.

### The expression of KIF2A in hypoxia-mDCs was modulated by HIF-1α

**Figure 4:**
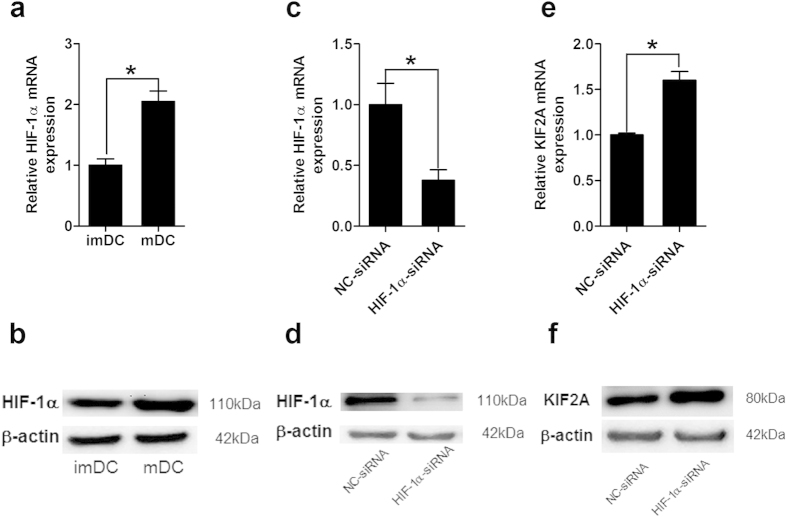
Knockdown of HIF-1α by specific siRNA up-regulated KIF2A mRNA expression in hypoxic mature DCs. (**a**,**b**) DCs were induced under hypoxia and matured by LPS. (**a**) Expression of HIF-1α mRNA was accessed by qRT-PCR. Results are shown as fold changes relative to mRNA levels in hypoxic imDCs and represent the average of three independent experiments. (**b**) Protein level of HIF-1α expression in imDCs and mDCs was detected by western blot with specific antibody. (**c**–f) Hypoxic imDCs were either electroporated with HIF-1α siRNA or negative control (NC) siRNA and LPS was added to induce DCs maturation. After 48 h, HIF-1α(**c**) or KIF2A (**e**) mRNA expression in mDCs was determined by qRT-PCR. The results are expressed as the fold mRNA levels of negative control siRNA treated DCs (arbitrarily defined as 1) of three independent experiments. Western blot was also performed to analyze the protein expression of HIF-1α (**d**) and KIF2A (**f**). *P < 0.05.

Since HIF-1α is a key mediator for hypoxia and it regulates a wide spectrum of biological processes and downstream targets^33^, we supposed the suppression of KIF2A in mDCs under hypoxia might also be mediated by HIF-1α activation. HIF-1α mRNA was detected in hypoxic imDCs; after LPS stimulation, HIF-1α expression in both mRNA and protein level became stronger in hypoxic mDCs (Fig. 4a,b), which was consistent with the findings by other groups in murine DCs^34^,^35^. To investigate whether the elevation of HIF-1α affected KIF2A expression in hypoxia-mDCs, specific siRNA targeting HIF-1α was used during DCs maturation under hypoxia. As expected, HIF-1α siRNA significantly reduced the mRNA and protein level of HIF-1α in hypoxic mDCs (Fig. 4c,d). KIF2A mRNA expression was increased by approximately 60% in hypoxia mDCs treated with HIF-1α siRNA compared to negative control siRNA treated group (Fig. 4e) as well as stronger expression of KIF2A protein was demonstrated by western blot (Fig. 4f). These results indicated the inhibition of KIF2A in mDCs under hypoxia was regulated by HIF-1α.

In summary, our results have shown that elevated expression of CD44 promoted the function of hypoxic mDCs to polarize Th2 response. Moreover, in hypoxia microenvironment, HIF-1α negatively regulated KIF2A expression, which further caused a decreased cell surface exposure of MT1-MMP, elevated expression of CD44 in mDCs. This suggested a novel mechanism by which hypoxia altered DCs functions through KIF2A. Such information provides important insights into understanding immune responses under hypoxia.

## Discussion

T-helper (Th) cells are major cytokine producers and are thus classified into Th1 cells and Th2 cells according to their cytokine production profile. Th2-mediated immune responses are primarily involved in atopic diseases, such as allergy and asthma^36^. Our previous findings showed hypoxic DCs polarized Th cells into a Th2 response with increased production of IL-4 and decreased IFN-γ^12^. Thus we explored its possible mechanisms in this study.

The CD44 expression on human monocyte-derived DCs has already been studied by other groups. During TNF-α-induced DC maturation, the expression of CD44 was up-regulated^22^,^37^. However, in our studies, when LPS was applied to stimulate DCs maturation, CD44 was found down-regulated in mDCs (data not shown). Such discrepancy might be interpreted by distinct signaling pathways adopted induced by various stimulus. Moreover, compared to normoxic mDCs, an enhanced expression of CD44 was found in hypoxic mDCs at both mRNA level and surface expression level, suggesting that CD44 play an important role in DCs function under hypoxia. Previous studies have proven that CD44 on mDCs was important for formation of clusters between DCs and T cells, and deficiency of CD44 in DCs led to decrease of T cell proliferation^24^. In addition, the conventional form of CD44 expressed in APCs has been shown to stimulate T cell proliferation^38^, suggesting that increased CD44 expression on DCs surface may modulate T cell stimulation, either through a direct effect on T cells or an indirect effect by inducing cytokine secretion in DCs. Consistent with our previous study, DCs matured in the presence of immobilized anti-CD44 mAb led to a skewed balance from Th1 to Th2 pattern. This indicated that the up-regulated CD44 in LPS-induced mature DCs under hypoxia could impair Th1 differentiation of naïve T cells through ligand binding.

CD44 mRNA was detected significantly up-regulated in hypoxic mDCs; and more interestingly, we found while surface CD44 expression on mDCs was enhanced by hypoxia, the secretion of soluble CD44 from mDCs was remarkably decreased. These results indicated hypoxia might regulate CD44 expression in DCs not only at transcriptional level, but also through trimming or shedding of CD44 from cell surface. The shedding is an event that could regulate the surface expression and soluble form of CD44. The proteases responsible for the shedding are mainly metalloproteinases. MT1-MMP, also known as MMP-14, was thought to be the enzyme responsible for the shedding. It could bind and cleave CD44, and over-expression of MT1-MMP enhanced CD44 shedding in the cells^25^. It has been extensively reported that MT1-MMP-dependent shedding of CD44 played an important role in lymphocyte physiology, such as the regulation of T-cell adhesion and transendothelial migration^39^. In our experiments, we found that the expression of MT1-MMP was reduced when DCs were cultured under hypoxia. When we further investigated whether the reduced expression of MT1-MMP contributed to the enhanced surface expression of CD44, indeed, it was observed that the cell surface expression level of CD44 was significantly increased in cells treated with anti-MT1-MMP. Meanwhile, blocking of MT1-MMP led to the reduced level of shed CD44 in the culture supernatants. Therefore, hypoxia could inhibit the surface exposure of MT1-MMP and, in turn, the reduced MT1-MMP contributed to the increased expression of CD44 in hypoxic mDCs.

Recent studies revealed that kinesins not only controlled cell mitosis but also were involved in cell migration and in the correct wiring of the brain^29^,^40^,^41^,^42^. In present study, we found that DCs highly expressed 5 kinesin member proteins. Most interestingly, hypoxia could significantly inhibit the expression of KIF2A in mDCs, which contributed to the decreased cell exposure of MT1-MMP at multiple levels. Furthermore, immunofluorescence microscopy analysis suggested that the distribution pattern of MT1-MMP was changed in KIF2A-knockdown mDCs. The surface-accessible MT1-MMP was clearly reduced and a lower level of MT1-MMP was found throughout the cytoplasm. Moreover, the silence of KIF2A resulted in a higher cell surface expression of CD44 in mDCs. These results indicated that KIF2A is crucially involved in regulating mDC functions and hypoxia could impact on the surface expression of MT1-MMP and CD44 via down-regulation of KIF2A.

HIF-1α is a key regulator of numerous molecular responses in hypoxic microenvironment, mediating a wide range of physiological and pathological processes^43^. As other groups have reported^34^,^35^, we also found HIF-1α expression was significantly increased during DC maturation by LPS under hypoxia. The results demonstrated that HIF-1α siRNA significantly reduced the expression of HIF-1α in hypoxic mDCs and KIF2A expression was suppressed by hypoxia through HIF-1α. Balaji Krishnamachary etc. showed HIF-1α was a regulator of CD44 expression in breast cancer cells under hypoxic conditions^44^. So HIF-1α might directly regulate the expression of CD44 in hypoxic mDCs or indirectly through KIF2A/MT1-MMP axis.

**Figure 5:**
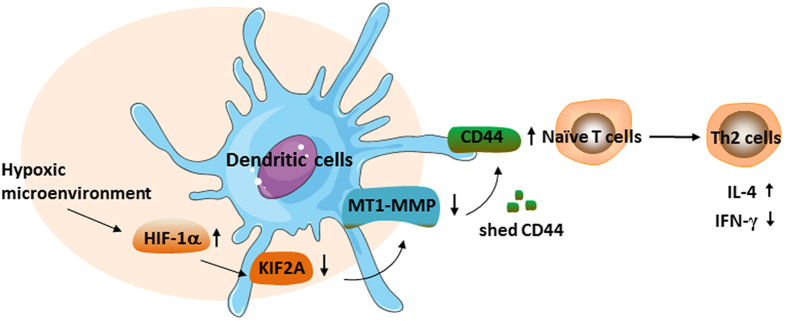
Schematic diagram depicting the mechanism of hypoxia-DCs skewed helper T cells toward a Th2 polarization. In hypoxic microenvironment, HIF-1α was activated and negatively regulated the expression of KIF2A. Down-regulation of KIF2A led to decreased cell exposure of MT1-MMP, and subsequently reduced the shedding of CD44 from DCs. Hypoxia was also found to directly regulate expression of MT1-MMP and CD44. Consequently, hypoxia-DCs promoted naïve T cells toward a Th2 polarization via increased CD44 ligand binding. Some elements (such as cells and molecules) of this figure were obtained from Powerpoint image bank of Servier Medical Art by Servier (http://www.servier.com/Powerpoint-image-bank) with small modifications, which is free for use and share, and is licensed under a Creative Commons Attribution 3.0 Unported License.

In conclusion, we described herein a new mechanism involved in hypoxia-DCs promoted Th2 response of naïve T cells with increased IL-4 and decreased IFN-γ production (Fig. 5). Hypoxia specifically down-regulated KIF2A, which led to decreased cell exposure of MT1-MMP, up-regulated CD44 in DCs. Also, hypoxia was found to directly regulate expression of MT1-MMP and CD44. Consequently, DCs were orchestrated toward a Th2 polarization from naïve T cells under hypoxia via increased CD44 ligand binding. Moreover, KIF2A expression was negatively regulated by HIF-1α in hypoxic microenvironment. Our findings therefore provide a novel insight into the role of hypoxia in programming DCs primed Th2 responses via a KIF2A/MT1-MMP/CD44 pathway and implicate that hypoxia might be a promising therapeutic approach in allergic diseases.

## Methods

### Generation of DCs

DCs were generated from human peripheral blood monocytes *in vitro* as previously described^12^,^14^. Briefly, monocytes were positively selected and enriched from PBMCs with anti-CD14 mAb-conjugated microbeads using the magnetic antibody cell sorting (MACS) system (Miltenyi Biotec) and their purity was confirmed by FACS (>93%). Immature DCs were generated by culturing monocytes in complete RPMI medium containing GM-CSF (1000 units/ml, R&D Systems) and IL-4 (500 units/ml, R&D Systems) for 5 days under hypoxia (1% O_2_) or normoxia (21% O_2_). 1 μg/ml LPS (Sigma-Aldrich) was added into imDCs to induce maturation for another 2 days. The use of peripheral blood from healthy volunteers was approved by the Medical Ethical Committee of Qilu Hospital, Shandong University. The methods were carried out in accordance with the approved guidelines and written informed consent was obstained from all volunteers.

### Real-time quantitative RT-PCR

Real-time quantitative RT-PCR (qRT-PCR) was carried out with LightCycler FastStart DNA Master SYBR Green I kit following the manufacturer’s instructions (Roche Diagnostics) and the data were analyzed by LightCycler software as described elsewhere^45^. The housekeeping gene β-actin was used as an internal control and the relative level of mRNA expression for target genes was normalized by β-actin. Primers designed for qRT-PCR are shown in Supplementary Table 1.

### Flow cytometry

DCs were stained with fluorescence labelled mAbs or corresponding isotype controls (Becton-Dickinson) and the expression of surface receptorswas detected by flow cytometry with a FACSCalibur flow cytometer (Becton Dickinson). CD11c was used for gating of DCs and the mean fluorescence intensity was analyzed by CellQuest software (Becton Dickinson).

### Enzyme-linked immunosorbent assay (ELISA)

The DC culture supernatants from different experimental groups were colleted, and shed CD44 (sCD44) concentration was determined by ELISA following the manufacturer’s protocol (Bender MedSystems).

### Th-cell polarization assay

Naïve CD4^+^ T cells were purified from human PBMCs using an immunomagnetic negative isolation system with MACS microbeads (Miltenyi Biotec; purity > 95% as determined by flow cytometry). Naïve T cells were cultured with DCs at a ratio of 5:1. IL-2 (50 U/ml; R&D Systems) was added on day 5 and phorbol myristate-acetate (PMA, 100 nM) and ionomycin (1 μg/ml; both from Sigma-Aldrich) was added on day 9 to induce cytokine secretion. On day 10, the supernatants were harvested and the production of IL-4 and IFN-γ was determined by using commercially available ELISA kits (R&D systems) following the manufacturer’s instructions.

### Immunofluorescence microscopy

DCs were fixed with 4% paraformaldehyde and blocked with TNB (0.5% blocking reagent; Boehringer Mannheim GmbH), followed by incubation with either anti- MT1-MMP LEM-2/15 (Millipore Biosciences) or isotype control Ab for 30 min at 37 °C. After washing in ice-cold PBS with 2% FCS, secondary Abs of FITC-goat anti-mouse IgG (Southern Biotech) were added and incubated for 30 min. Cell nuclei were stained using DAPI. The location of MT1-MMP was detected by immunofluorescence microscopy (Olympus IX81) and its relative immunofluorescence intensity was qualified using Image J software.

### Western blotting analysis

Total proteins prepared from DCs were separated on a SDS polyacrylamide gel (SDS-PAGE) and transferred to a polyvinylidene fluoride (PVDF) membrane (Amersham Pharmacia Biotech). Following blocking with 5% nonfat milk in TBS-T (Tris buffered saline plus 0.05% Tween-20), the membrane was incubated with anti-MT1-MMP (Millipore Biosciences), anti-KIF2A (Abcam) or anti-HIF-1α (Abcam) at 4 °C overnight. Then, the membrane was washed 4 times, 5 min each in TBS-T before incubating with horseradish peroxidase (HRP)-conjugated goat anti- rabbit or mouse IgG (Jackson Immuno Research Laboratory). Anti-β-actin mAb (Abcam) was used as an internal control. Protein bands were visualized with enhanced chemiluminescence detection system (ECL, Amersham Pharmacia Biotech) following the manufacturer’s instructions.

### Sample preparation and microarray analysis

Total RNA samples from normoxic and hypoxic mDCs were prepared and processed as recommended by Affymetrix (Affymetrix Inc.) and the transcriptional gene expression profiling was further detected following the Affymetrix GeneChip Expression Analysis protocol, which has been described previously^12^. The results were quantified and analyzed using the Genchip Microarray Suite V5.0 software program (Affymetrix Inc.). Differential expression of transcriptional profile of kinesin family members was assessed by pairwise comparisons of hypoxic mDCs to normoxic mDCs, where an average difference >500 indicated detectable expression and differential expression required a more than two-fold change in mRNA levels^46^.

### RNA interference

siRNA duplexes targeting KIF2A or HIF-1α and negative control siRNA were transfected into imDCs by electroporation using human dendritic cell transfection program according to the manufacturer’s protocol (Amaxa biosystem) as previously described^14^. 1 μg/ml LPS was added to induce DCs maturation. After 48 hours, the transfected cells were harvested and target gene expression was analyzed by qRT-PCR, western blotting and/or immunofluorescence microscopy, respectively.

### Co-immunoprecipitation experiments

Cells were lysed in 0.5 ml of NETN lysis buffer (100 mM NaCl, 1 mM EDTA, 20 mM Tris, pH 8.0 0.5% Nonidet P-40) containing complete protease inhibitor mixture (Roche Diagnostics Ltd), 10 mM NaF, and 10 mM-glycerolphosphate. Cell lysates were incubated with 2 μg antibodies against KIF2A or MT1-MMP at 4 °C for 5 hours. Subsequently, protein A+G agarose beads were added and mixture was rotated for 1 hour at 4 °C. After three wash steps in lysis buffer, bound proteins were eluted by boiling in SDS-sample buffer and detected using western blotting.

### Statistical analyses

Results are expressed as means ± SD. The differences between experimental grous were analyzed using Student’s *t*-test and *P* values of <0.05 were considered statistically significant.

## Additional Information

**How to cite this article**: Yang, M. *et al.* Increased expression of surface CD44 in hypoxia-DCs skews helper T cells toward a Th2 polarization. *Sci. Rep.* **5**, 13674; doi: 10.1038/srep13674 (2015).

## Supplementary Material

Supplementary Table 1

## Figures and Tables

**Table 1:** Transcriptional profile of kinesin family members in mDCs.

Gene title	Gene Symbol	Detection (H-mDCs)	Detection (N-mDCs)	
Kinesin family member 1A	KIF1A	A	A	
kinesin family member 1B	KIF1B	P	P	
kinesin family member 1C	KIF1C	A	A	
kinesin heavy chain member 2	KIF2A	P	P	
kinesin family member 2C	KIF2C	A	A	
kinesin family member 3A	KIF3A	P	P	
kinesin family member 3B	KIF3B	P	P	
kinesin family member 3C	KIF3C	A	A	
kinesin family member 4A	KIF4A	A	A	
kinesin family member 5A	KIF5A	A	A	
kinesin family member 5B	KIF5B	P	P	
kinesin family member 5C	KIF5C	A	A	
kinesin family member 9	KIF9	A	A	
kinesin family member 11	KIF11	A	A	
kinesin family member 12	KIF12	A	A	
Kinesin family member 13A	KIF13A	A	A	
kinesin family member 13B	KIF13B	A	A	
Kinesin family member 14	KIF14	A	A	
kinesin family member 17	KIF17	A	A	
kinesin family member 18A	KIF18A	A	A	
kinesin family member 20A	KIF20A	A	A	
kinesin family member 21A	KIF21A	A	A	
kinesin family member 21B	KIF21B	A	A	
kinesin family member 22	KIF22	A	A	
kinesin family member 23	KIF23	A	A	
kinesin family member 24	KIF24	A	A	
kinesin family member 25	KIF25	A	A	
Kinesin family member 26A	KIF26A	A	A	
kinesin family member 27	KIF27	A	A	
Kinesin family member C1	KIFC1	A	A	
kinesin family member C2	KIFC2	A	A	
kinesin family member C3	KIFC3	A	A	

A, absent expression; P, positive expression.

H-mDCs, hypoxic mature DCs; N-mDCs, normoxic mature DCs.

**Table 2:** Relative expression of kinesin family members in mDCs.

Gene title	Gene Symbol	Detection (H-mDCs)	Log Ratio	Change	Detection (N-mDCs)
kinesin family member 1B	KIF1B	P	0.1	NC	P
kinesin heavy chain member 2	KIF2A	P	−1.4	D	P
kinesin family member 3A	KIF3A	P	−0.2	NC	P
kinesin family member 3B	KIF3B	P	−0.9	NC	P
kinesin family member 5B	KIF5B	P	−0.2	NC	P

P, positive expression; NC, no change; D, decreased.

H-mDCs, hypoxic mature DCs; N-mDCs, normoxic mature DCs.
