# Supplementary material for: Increased expression of surface CD44 in hypoxia-DCs skews helper T cells toward a Th2 polarization
Source: Sci Rep. 2015 Sep 1;5:13674. doi: 10.1038/srep13674 (PMC4555176; doi:10.1038/srep13674)
Supplement: Supplementary Table 1 [file srep13674-s1.doc]

**Increased expression of surface CD44 in hypoxia-DCs skews helper T cells toward a Th2 polarization**

Meixiang Yang^1,7^, Yanguo Liu^2,7^, Guangwen Ren^3^, Qianqian Shao^1^, Wenjuan Gao^1^, Jintang Sun^1^, Huayang Wang^1^, Chunyan Ji^4^, Xingang Li^5^, Yun Zhang^6^, Xun Qu^1^

^1^Institute of Basic Medical Sciences, Qilu Hospital, Shandong University, Jinan, 250012, Shandong, China.

^2^Department of Medical Oncology, Qilu Hospital, Shandong University, Jinan, 250012, Shandong, China.

^3^Department of Molecular Biology, LTL255, Washington Road, Princeton University, Princeton, New Jersey 08544, USA.

^4^Department of Hematology, Qilu Hospital, Shandong University, Jinan, 250012, Shandong, China.

^5^Department of Neurosurgery, Key Laboratory of Neuro-Oncology and Immunology, Qilu Hospital, Shandong University, Jinan, 250012, Shandong, China.

^6^The Key Laboratory of Cardiovascular Remodelling and Function Research, Chinese Ministry of Education and Chinese Ministry of Health, Qilu Hospital, Shandong University, Jinan, Shandong 250012, China.

^7^Meixiang Yang and Yanguo Liu contributed equally to this work.

*Corresponding author

Xun Qu, Institute of Basic Medical Sciences, Qilu Hospital, Shandong University, #107 West Wenhua Road, Jinan 250012, Shandong, People's Republic of China. Tel: +86-531-82169251; Fax: +86-531-82169251; E-mail: quxun@sdu.edu.cn

**Running title**: CD44/MT1-MMP/KIF2A modulates hypoxia-DCs function

**Supplementary Table 1.** Primer pairs used for real-time quantitative RT-PCR

| Gene | Primer sequences |
| --- | --- |
| CD44 | Forward 5'-CCTCCAGTGAAAGGAGCAGCAC-3' |
|  | Reverse 5'-GTGTCTTGGTCTCTGGTAGCAG-3' |
| MT1-MMP | Forward 5'-CCGATGTGGTGTTCCAGACA-3' |
|  | Reverse 5'-TGGCCTCGTATGTGGCATACT-3' |
| KIF1B | Forward 5'- AGGAGGAAGCTCACTGTGGA-3' |
|  | Reverse 5'-CAAGACAACTGGCAAAGCAA-3' |
| KIF2A | Forward 5'-GCCTTTGATGACTCAGCTCC-3' |
|  | Reverse 5'-TTCCTGAAAAGTCACCACCC-3' |
| KIF3A | Forward 5'-TGGCAGCTAAAATGTGTTGC-3' |
|  | Reverse 5'-CTGTCTTTGGCCTTGCTTTC-3' |
| KIF3B | Forward 5'-CCCCAATAGACCCCCTTTTA-3' |
|  | Reverse 5'-CGGGAAATAATCCAGCGTTA-3' |
| KIF5B | Forward 5'-TGCGGAACACTATTCAGTGG-3' |
|  | Reverse 5'-ATTGCGGTTGCTGGTTTATC-3' |
| HIF-1α | Forward 5'-TGCTTGGTGCTGATTTGTGA-3' |
|  | Reverse 5'-GGTCAGATGATCAGAGTCCA-3' |
| β-actin | Forward 5'-TTGCCGACAGGATGCAGAA-3' |
|  | Reverse 5'-GCCCGATCCCACACGGAGTACT-3' |
